# Establishing stability: exploring the meaning of ‘home’ for women who have experienced intimate partner violence

**DOI:** 10.1007/s10901-016-9511-8

**Published:** 2016-05-17

**Authors:** Julia Woodhall-Melnik, Sarah Hamilton-Wright, Nihaya Daoud, Flora I. Matheson, James R. Dunn, Patricia O’Campo

**Affiliations:** 1 Centre for Research on Inner City Health, The Keenan Research Centre in the Li Ka Shing Knowledge Institute, St Michael’s Hospital, 3rd Floor, 30 Bond Street, Toronto, ON M5B 1W8 Canada; 2 Department of Public Health, Faculty of Health Sciences, Ben-Gurion University of the Negev, Beersheba, Israel; 3 Dalla Lana School of Public Health, University of Toronto, Toronto, ON Canada; 4 Department of Health, Aging and Society, McMaster University, Hamilton, ON Canada

**Keywords:** Experiences of home, Housing stability, Intimate partner violence, Ontological security, Women and housing

## Abstract

There is evidence that involuntary housing instability may undermine health and well-being. For women who have experienced intimate partner violence (IPV), achieving stability is likely as important for other groups, but can be challenging. Through our analysis of 41 interviews with women who have experienced low income and IPV, we argue that definitions of housing stability are multifaceted and for many centred on a shared understanding of the importance of creating an environment of “home”. We found that obtaining housing that satisfied material needs was important to women. However, in asking women to define what housing stability meant to them, we found that other factors related to ontological security and the home, such as safety, community, and comfort, contributed to women’s experiences of stability. Through our discussion of the importance these women placed on establishing stable homes, we argue that future research on women’s experiences with housing stability and IPV should include definitions of stability that capture both material security and women’s experiences with building emotionally stable homes.

## Introduction

Recent studies have examined the relationship between women’s experiences of violence and housing instability (Baker et al. 2010; Daoud et al. 2016; Jategaonkar and Ponic 2011; O'Campo et al. 2015; Pavao et al. 2007; Rollins et al. 2012). Researchers often rely on definitions of stability that focus on the material conditions (e.g. affordability and permanence) associated with maintaining housing. Ponic et al. (2011) and Kushel et al. (2006) define stability as few or infrequent moves and adequate, affordable, and suitable (e.g. good quality and large enough for family size) housing. These studies focused primarily on material and physical aspects of housing stability neglecting psychosocial dimensions of housing such as feelings of safety, security belonging, and attachment to home, all important aspects of women’s experiences of personal and housing stability (Daoud et al. 2016; Despres 1991; Dunn 2002; Dunn et al. 2004a, b; Dupuis and Thorns 1998).

To understand women’s complex definitions of stability, we distinguish between the house as a physical place which requires material maintenance and the home as a place to which people ascribe emotive meaning. Place attachment theory postulates that people can assign deep emotional meaning to physical spaces which results in bonds with places (Manzo and Devine-Wright 2014). Using a phenomenological lens, Seamon (2014) argues that physical environments cannot be understood as separate from the people who occupy them. In other words, physical places are more complex than bricks, mortar and furniture. The experiences of occupants and their ascribed meanings are interwoven with the place as a physical space (Seamon 2014). Emotions attached to places can be both positive and negative. In studying low-income public housing tenants’ perceptions on forced housing relocations, Manzo (2014) found that some tenants felt security contributed to affordability, social ties, and the availability of services, whereas others expressed ambivalence or negativity stemming from stigma, poor housing conditions, and social problems. Regardless of positive or negative views of home, the majority of residents were still socially and/or emotionally attached to their homes and felt disrupted by forced relocation (Manzo 2014).

Given that many women experience forced relocation when they leave an abusive relationship it is worth exploring their experiences of housing instability and ‘home’. To our knowledge, this is the first study to address feelings of home specifically in women who experienced IPV. Head (2005) investigated feelings of home among lone-mothers living in social housing and who received income support in England. He found that women’s views of home were impacted by whether they had insider status in the neighbourhood, social networks, opinions of neighbourhood crime and racism, and their experiences as mothers. Our study will specifically focus on how women who have experienced IPV in Canada define stable housing. Broad definitions of housing stability, although useful for understanding general housing trends, do not incorporate women’s personal definitions and realities. Considering the lived-experience of women is important to improving equity, as it allows for an understanding of the dynamics of an issue. When definitions are built using lived-experience, in combination with quantitative measures, researchers are able to gain a deep understanding of an issue while monitoring change over time (Popay et al. 2008).

### Conceptualizing stability and home

In earlier work, Saunders (1989) argued that home ownership was a source of ontological security. Studies by Hiscock, et al. (2001) and Dunn (2013) have reinforced and extended this perspective to include rental housing and to integrate notions of the meaning of home and the construction of self-identity (Dupuis and Thorns 1998). Giddens (1991, 243) defines ontological security as “a sense of continuity and order in events, including those not directly within the perceptual environment of the individual.” A deficit in one’s sense of order or routine can create existential anxiety or worry about what is unknown and the inability to protect oneself from perceived “future threats and dangers” (Giddens 1991, 39). Giddens (1991, 242) also theorizes that individuals engage in a “reflexive project of the self”, which is “ordered by self-narratives” that link one’s past to the present and to the future (the latter Giddens calls “colonization of the future”). This conceptualization is consistent with other work on identity and self in social psychology, such as the notion of ‘possible selves’ by Markus and Nurius (1986) and Segal et al.’s (2001) research showing that people construct self-narratives of possible futures that affect them in real, material ways. Indeed, Segal et al. (2001) show that people of different socio-economic status construct narratives of the possible futures selves they can imagine for themselves that are very different and significantly affect their life chances. Ultimately, the self-narratives that comprise the reflexive project of the self play an integral role in forming an individual’s ability to create order and stability in daily life, and, by extension, ontological security (Giddens 1991).

Recent conceptual extensions of Giddens’ theoretical approach applied to housing have clarified the role of ontological security in understanding the importance of home (Dunn 2013). These arguments have extended Saunders’ (1989) claim that owner-occupation is the only form of tenure that is capable of supporting ontological security. Indeed, it is very plausible that with adequate legal protections, other forms of tenure, including market rental, can support ontological security (Dunn 2013). In discussing the home within the neoliberal era, Dunn (2013) argues that the importance placed on the home as a material representation of self has bound it to individuals’ experiences of ontological security. In employing the metaphor of the “dream home,” Dunn (2013, 190) articulates the capacity for idealized notions of home to serve as a vision of one’s future, alluding to the importance of colonization of the future and ontological security in building identity and establishing control and order in life. Housing and home have the capacity to provide security, resilience, and enhance wellbeing (Dunn 2013). In this sense, the home is intricately connected to one’s reflexive project of the self which in turn suggests that housing pathways (Clapham 2005) play a central role in experiences of ontological stability. In viewing housing through a ‘pathways’ approach, we argue that it is necessary to think about housing situations as fundamentally woven through an individual’s life course, and pay close attention to the meanings attached to the home, the relationship with other life events, and an individual’s biography (Clapham 2005). It follows that when individuals are unable to generate a narrative of housing stability in their narratives of the future, existential anxiety may result. Conversely, if a person’s housing situation is chronically unstable, even if it is due to the behaviour of another member of the household, and the person leaves that housing situation, his or her experience of subsequent housing situations will be through a lens of seeking stability not only in the material circumstances of housing, but also in the narratives of the self that can be created to gain ontological security.

In her work, Padgett (2007) describes home as a mechanism for contributing to ontological security for persons who have experienced psychiatric symptoms and homelessness. In doing so, she connects the concept of home to ontological security, using Shaw’s (2004) description of housing stability. Specifically, Padgett (2007) argues that for this population, stabilization of housing provided new experiences of routine, control and the opportunity to engage in development of self. There may be reason to believe that other groups facing chronic housing instability, like women experiencing intimate partner violence (IPV), that is subsequently stabilized, will have similar experiences.

Most importantly for the current study, women leaving violent households will experience changes in housing stability and arguably ontological security. While on the one hand, women who leave situations where their partners have used housing stability as a tactic of abuse may experience an increase in ontological security, the experience of transition housing may, due to its inherent instability, delay women’s abilities to achieve a state where their housing is a major contributor to ontological security.

### Intimate partner violence and housing instability

Women who have been living without a feeling of ontological security are in a sense experiencing displacement from their homes (Brickell 2012). Robinson (2008) argues that IPV has long-term impacts on an individual’s capacity to secure and maintain stable homes. She states that along with the grief and psychological trauma experienced by women facing IPV, there is also an alienation that evolves from “being without the corporeally geographically and socially orienting place of home” (Robinson 2008, 98). In this sense, homelessness is a “felt-experience of displacement” (Robinson 2008, 98) and housing instability contributes to the trauma women experience when living in or leaving violent households. Broadly speaking, the literature on ‘home’ distinguishes between a house as a physical location which provides material stability and a home as a meaningful place that fulfills the social, emotional, cultural or psychological needs of its members (Dam and Eyles 2012; Manzo 2003; Vandemark 2007; Warrington 2001). In Robinson’s (2005) study of the displacement of young homeless people, she discusses the role of therapeutic places as a means to rediscover positive relationships of self (Robinson 2005); therapeutic places provide “stable accommodation and a conduit to an embodiment of being-at-home” (Robinson 2005, 57).

In their study of homeless women in the UK, Tomas and Dittmar (1995) defined home as a place where one feels safe and secure. They found that stably housed women were able to clearly articulate the differences between houses and homes, whereas only 25 % of the homeless women studied were able to clearly articulate the difference between the two concepts. This study illustrates the variations in meaning of home for different groups of women, suggesting the need to understand stability from a variety of different vantage points. Tomas and Dittmar (1995) call for a closer investigation of the meaning of home for different populations of women who experience housing instability. Mallet suggests there is a gap our knowledge about the interconnection between gender and home, specifically as it relates to home as a therapeutic, rather than oppressive, place for women (Mallet 2004). This paper explores the conceptualization of ontological security and ‘home’ among women who have recently experienced IPV. The aim of this paper is to describe and discuss how women who have experienced IPV define stable housing and to connect these definitions with their experiences of ontological stability.

## Methods

The data in this paper came from a larger qualitative study designed to explore the relationship between housing instability and health among women who had recently experienced low-income and IPV (see Daoud et al. 2016; Matheson et al. 2015; Minh et al. 2013; O'Campo et al. 2015; Velonis et al. 2015). In this larger study, we asked women to define stable housing using their own words. The question that guided the analysis for this paper was: how do low-income women who have experienced IPV define stable housing? In order to address this question we analyzed data from interviews with 41 women from five urban and non-urban areas in Ontario, Canada. The sample was recruited through a network of organizational contacts, including social housing authorities, women’s centres, and social services agencies. These organizations displayed and distributed study flyers. Additionally, staff at these organizations were asked to inform potential participants of the research.

Potential participants were asked to phone our study line. During the phone conversation each woman was screened for eligibility: aged 25–60, experienced IPV within the past 5 years, and were residing in social, transitional, or market rental housing (see O'Campo et al. 2015). Forty-one women were interviewed in their homes. The women were asked questions, using semi-structured interview guides, to elicit their experiences of health, housing and violence throughout their life courses. Each interview lasted approximately one to 2 h and was tape recorded for accuracy. This study received approval from St. Michael’s Hospital’s Research Ethics Board. To maintain confidentiality, participants were assigned pseudonyms.

After transcribing each of the tape recorded interviews, the process of open coding was conducted by the research team. Inter-rater reliability (valuation of consistency amongst coders) was assessed (Gwet 2012). Coding involved carefully reading each interview and labeling each passage. This process involved meeting frequently to discuss the coding process (see Daoud et al. 2016). The open coding process revealed a list of codes and themes that were inserted into NVIVO qualitative analysis software. In order to generate the data for this paper, two team members (JWM, SHW) analyzed the data within the perceptions and experiences of stability codes. Particular attention was given to women’s experiences and definitions of housing stability. The two team members reviewed and summarized data on housing stability independently and then met at frequent intervals to discuss emergent themes and to reach a consensus on the construction of the themes for this paper.

All of the women in the study were living in social housing (48.8 %), market housing (31.7 %), or transitional housing (19.5 %) at the time of the interviews. Roughly half of the women interviewed were 40 years of age or younger (48.8 %). The majority of participants were born in Canada (90 %) and were White (53.7 %) or Black (14.6 %). Just under half (41.5 %) of the participants were living with dependent children under the age of 18 and most were either separated/divorced (41.5 %) or single (51.2 %). The majority of the women were unemployed (39 %) or receiving provincial disability benefit payments (41.5 %). A large proportion (58.5 %) had educational credentials beyond the secondary level. Despite this, 73.2 % reported incomes below $20,000. All of the women interviewed had experienced some form of IPV in the past 5 years. The majority of women were no longer living in abusive relationships. The majority of these women reported experiencing both physical and verbal violence, whereas only 13 % had experienced verbal violence alone. Two thirds reported that their abusive partner’s violent behavior constrained their actions. This indicated that the majority of abusive partners exhibited controlling behaviour.

The majority of municipalities in Ontario have emergency shelters for women who are in need of a place to stay after leaving a violent household. In addition to this, Ontario’s social housing legislation (the Housing Services Act) contains the Special Priority Policy. This requires that any individual who is leaving an abusive household and qualifies for low-income housing subsidies be placed at the top of the social housing waiting list (Special Priority Policy Research Taskforce 2012). This may help low-income women who have experienced IPV obtain material housing stability; however, our research shows that other components of stability were important to women.

## Findings

We asked women about their housing histories. This allowed us to gain a better understanding of housing careers and trajectories. In order to better comprehend women’s definitions of stable housing, we also asked them to define stability in their own words, including what stable housing meant to them. We sought to elicit women’s own definitions and experiences of housing stability. As women described elements of their personal experiences across a variety of current and past housing situations, a number of themes arose. In this section, we present these themes as we work toward a meaningful definition of stability for women who have experienced IPV which can be used in future research to better assess whether or not women experience housing stability when leaving and living in abusive relationships.

Other papers using the data from this study address instability and decisions to leave, stay with and return to abusive partners (Daoud et al. 2016; Velonis et al. 2015), whereas this paper specifically addresses stability and women’s views on stability. We recognize that women may continue to experience instability after leaving their partners (O'Campo et al. 2015); however, research shows that stable housing can promote stability in other areas of life (e.g. substance use, mental health, etc.) (Tsemberis et al. 2012), suggesting that securing stable housing may help women generate stability in other areas of life.

Most of the women interviewed for this study had experienced a long history of residential instability. Despite this however, they did know what stable housing meant to them and had made one or more attempts to achieve stability throughout their life courses. Achieving housing stability was a constant goal for these women, as Natasha described:

Stable housing…[is] consistent, I suppose, always there, affordable….stable, not sure I have lived in any stable housing for a long time. So I mean, I have an idea of what it is, and I am looking to get towards it.

Shortly after, and long after, leaving their abusive partners, women struggled to achieve and maintain their ideal vision of housing stability. However, some women in our study were on the path toward their goals of achieving stable homes. We found that housing stability includes material and structural stability. However, for many women, this was not enough. Rather, additional characteristics of housing stability were important to these women.

### Material stability

The majority of women described the importance of affordability and permanency in their definitions of housing stability. A selection of women’s words are reported below.

#### Permanence

Traditional definitions of housing stability involve the concept of permanency (Kushel et al. 2006; Ponic et al. 2011). Many of the women defined periods of housing stability as times in their lives when they had the ability to maintain housing for a significant length of time. The ability to remain in one place for a period of time was valued by women and their children as it gave them the opportunity to connect with their community and establish trusting relationships. This allowed them to rebuild and form social networks. Abby described how important stable housing was to her and her children:

I think this house was the best thing we have done in a while, it was the best decision, it’s the place we’ve been almost the longest of everything, of any place and though the odd time I think of moving, it would be interesting but I don’t think so. I think this is the house we will be in for a long time because my kids don’t want to move, they love the friends they have across the street and they love our neighbourhood.

Rosie elaborated on this while reflecting on her past housing experiences:

[In my past, housing experiences] were uniformly bad. I just never felt a sense of security, permanence… here I have permanence, I’m secure. I didn’t feel that in all that time. I didn’t feel like it was mine, as well as my partner’s. I just, I didn’t feel, just no security. You know? That nothing wasn’t going to happen, that I wouldn’t be sleeping on a friend’s couch the next day, which I have done a lot of. That sort of thing, so, yeah.

Rosie felt as if her housing was insecure because of the violence she had experienced. She worried about having to leave her home at a moment’s notice. For Rosie, wondering whether or not she would be able to continue to stay in her house, coupled with frequent periods of leaving her home, resulted in feelings of instability. The connection between permanency and stability was discussed by many women in this study, suggesting that common measures of stability resonate with women who have experienced IPV.

#### Financial independence and affordability

Financial independence and affordable housing were both mentioned multiple times as imperative to stability. Women sought homes that were affordable in general; however, they also described the importance of being financially independent or self-reliant. Having their own home, or a place to call their own, was an aspect of independence that women mentioned during periods of stable housing. Elizabeth said:

When I had my own house and stable housing, I wasn’t relying on them for my housing or for my well-being so, I think the only times I have had bad relationships is when I was living with them, and they were kind of calling the shots. Whereas when I was paying all my own bills and taking care of myself I never had a problem with relationships.

Randi also reflected upon a time when she had stable housing. During this time, her work provided her financial independence:

I didn’t have to worry about a thing. I was working usually three jobs and when I had the restaurant for seven years, that was one hundred and twenty hours a week, plus keeping up with the clean house and taking care of two children. Nothing put me down. I think the kids really had me, when I had the kids with me, that I didn’t have time to go on a self-pity trip. I didn’t have time, because I had so many activities to go on.

Although Randi was constantly occupied with work, household duties, and childcare responsibilities, she felt a sense of purpose and wellbeing which she derived from meeting her family’s financial needs.Victoria lived in social housing and discussed the importance of material stability:

Love it. It’s great! Even the market rent here is 700 dollars so I can afford it. It’s a three bedroom, it’s on a good street, it’s a really nice community, there’s a good school up the street, so, I’m happy…I’m happy that I’m here, like I can breathe out here, like before I couldn’t breathe cause I’d think, that’s where all the violence happened…and it gives you the opportunity for you and your children to be in a safe place, so hopefully it’s where you can build and heal. I feel I can heal here…it’s a stepping stone.

Victoria described being able to feel independent. She attributed this to the affordability of her subsidized housing. She chose to physically move away from the city where she experienced violence and this allowed her to begin to rebuild her life. She was able to do this because she was able to access safe, affordable housing and achieve financial independence.

### Emotional stability

Even when women had material stability, IPV contributed to feelings of instability, Zoe described this:

So I would say with my ex, interestingly enough it was more unstable. Which I always found interesting because in a sense we had more money and but at the same point if you are with someone in an unstable relationship, right? And that’s sort of the eye opener you know? It’s like, pros and cons, you know? When you kind of look at it. I could stay and have more money and probably be more respected because we are like a family but at the same time there is that dysfunction.

The women in this study noted the importance of material aspects of housing stability; however, they also included other components such as safety, consistency, and comfort in their definitions of home. In other words, the material aspects of housing stability continued to be important, but their experiences with violence provided them with additional criteria that needed to be met before they could feel stable in their homes.

#### Safety

Many women reported that stable housing involves feeling safe from violence. For these women, stable housing provided a sense of control. Women reported that they felt that the presence of surveillance cameras and monitored surroundings in their current residences provided them with a greater sense of security. Claudia stated:

I feel like safe here. I feel that, I don’t have anybody that can tell me to leave. And, I don’t have to put up with anybody’s crap [laughs]. Cause it’s just me and my daughters, so. I’m very grateful that I got into [this] housing.

Claudia refers not only to her personal safety, but the safety of her children. She viewed safety in a relational sense, seeing her connection to her kids and disconnection from her abusive partner as offering a sense of control, stability, and security.

Safe housing was often viewed as a mechanism for increasing stability in other areas of life. Zoe stated:

Stable housing to me is housing that I can afford and that is in a safe place that is going to benefit me and my family. That it’s not just that its affordable in a sense that if I’m working or not working that I’ll be able to stay there, it’s stable in that we are going to feel safe. You know? Like all around the environment and the people around and that we will be able to stay there and kind of progress….And that it’s going to improve our situation as well like it’s not going to make it worse and that’s health wise socially, emotionally like everything

Living in safe accommodations is important to women and speaks to their desire to heal and rebuild their lives after the lack of safety created by living with and/or leaving violent relationships.

#### Consistency

Women’s definitions of what stable housing meant to them illustrate the importance of forming predictable or consistent life routines. For example, Rosie defined stability:

It means living in one place for an amount of time, not moving from pillar to post, like I had been doing. Stability. Just for me, it means living in a normal atmosphere, which is consistent, has consistency, has routine. All of those things. That everything is financially looked after, regular….like regular folk. You know, to do homework, my daughter going to school. Stability. Just not that chaos. Talking things out. Stability is a whole gambit of things.

Rosie defined stability as absence from chaos. In this sense, achieving stable housing could be seen as a contributor to ontological security as it was associated with establishing routine. This mirrors Dunn’s (2013) assertion that having a sense of place assists in establishing order. Routine provided a reprieve from chaos which was viewed as a stabilizing factor by the women.

#### Comfort

In addition to safety as an element of home, other women included home in their definitions of stable housing as being a place of comfort and peace. This connects with experiences of violence, as all of the women interviewed lacked calm while living with their abusers. In her definition, Lacey stated that stable housing is:

A place of peace. Comfort and security… Having your own space, I like my own space, so it’s peaceful that way. Away from worrying about the drinking or the nattering that goes on. I’m tired of that.

Suzie defined home:

Somewhere like your own home. Like somewhere comfortable. Somewhere you feel safe.

Women’s definitions stability involved hard or material concepts (e.g. permanency and affordability) comfort and affordability but they also included soft measures (e.g. comfort and consistency) that are cited as contributing to ontological security (Dunn 2013). Additionally, definitions also included safety which may have resulted from their past experiences with violence and abuse, suggesting the importance of the concept of safety in constructing meaningful definitions of stability for women who have experienced IPV.

### Stability as key to moving forward

All of the women in this study had experienced housing instability as a result of living in and leaving violent housing situations and were in different stages of rebuilding their lives. Housing remained an important foundation for women in this regard and their descriptions highlight how housing is a central part of this progression for them. In analyzing women’s definitions of stable housing, a unique perspective emerged. For these women an important element of stable housing was that it provided a means for them to achieve a foundation upon which to start again, to grow and “feel free and plan for the future in.” Women felt that stable housing should not “take everything they have” and it should create a stable situation. Zoe was in the process of rebuilding her life and described the importance of stable housing:

With more [stability], if the person wanted to have goals in their life and just keep going ahead and move on, and have things to do for themselves one could progress.

Zoe suggested that stable environments should allow people to progress forward and grow. She discusses housing stability as a mechanism that can assist women with making progress in their new lives.

As many of the women in this study struggled with addiction and mental health issues, stability also meant living in a place that does not put them at risk of being “triggered” by “certain areas or people who are on drugs.” Naomi lived with a significant mental health problem and addiction issues and she described how, after leaving a violent relationship, social housing assisted her with independence and regaining her health:

…[Social] housing really helped me a lot. To get healthy, and to be healthy and to be a productive member of society. To be well.

For Naomi, stable housing helped her gain a positive sense of self as she began the process of coping with her addiction and mental health issues. To her, stability meant finding a place to live where she could grow and rebuild her life.

Although permanency was an important component of stability for women, some women did express that feelings of stability could be experienced in temporary housing situations, such as transitional housing, and women’s shelters. They argued that others’ warmth, compassion, and access to supportive services contributed to these feelings of stability. Randi, discussed these feelings of stability while living in temporary housing:

[Stable housing] means it’s a safe environment for me to be in and affordable. I have less worries. I am very grateful that I have this apartment for what I pay for it, because I was in no position to be paying market rent at the time. Just what it has to offer. It has a lot of qualities, with counseling and trying to get you back in the outside world. It’s more than just housing. It’s more than just a roof over my head. This place has provided for me. I’m not sure all housing (does), because this is a place for battered women, it’s a counseled place. They’ve taught me an awful lot and I’ve done a lot of growing here, you know? Because when I think about when I came here, I wasn’t healthy at all. I was very weak, fragile and I suppose if I was in a different place, just a regular building, living with mixed and matched, then I could have been dragged down.

For Randi, living in transitional housing which was geared towards women who experienced violence provided her with the support she needed to regain stability in different aspects of her life. She felt as if this experience was about more than a roof over her head, as it enabled her to process traumatic experiences. She recognized how important stability was to her health and healing. In this case, stable housing was a temporary, yet therapeutic, space.

Women who had achieved stable housing were asked how their housing affected their children’s health and well-being. Many of these women included their children in definitions of housing stability, arguing that stable housing situations meant that their children could feel secure in their routines, reside in safe neighbourhoods, and build social connections with peers. Lynne described the role that transitional housing played in her children’s lives. She noted that the housing was not ideal, but it did provide a sense of home that improved her children’s sense of wellbeing and security:

Well now that they are in a safe home and secure housing well as safe as it could be they are different. They are more open to have friends over and to make friends and they are most secure with their routine because they know that they are not going anywhere there are no hazards… When I used to live at the place that was a lot more insecure in regards to who would they would meet outside and that kind of thing. We would never go out late at night or if I did go out at all and my daughter was babysitting I used to have to kind of lock the door, ‘don’t open it for anybody just open it for me’ that kind of thing and I wouldn’t let them out late at night either… To give them that feeling of safety and security I think that is one of the first steps to letting them really begin their lives again. And I have seen the changes it made for me and my kids, It may not be ideal but still. This was the first time where I my son actually called where we live home. When we first moved in he’s like “when are we going home mommy?” and then when we would come, he would see the building and he said “this is our home” and this is the first time he ever called a place home and he feels relaxed enough in it.

Many of the other mothers who had children living at home also discussed their children in definitions of stability. For many of these women, their relationship with their children was their most important relationship. Being able to feel personal safety and security, as well as being able to feel like their children are safe, contributed to a feeling of home for both themselves and their children. Housing played a key role in assisting women with rebuilding ontological security, as it facilitated the building of trusting relationships and assisted women with gaining a sense of permanency, routine, and control. They felt as if their housing situations were stable because they felt safe letting their children make friends. These women were able to feel safe in their homes and neighbourhoods. For many women, knowing that their children were safe and happy contributed to housing stability.

Some of the women described stability as a continuum. This meant that throughout their lives, they had experienced varying degrees of both instability and stability. For them, the journey along this continuum of stability was nonlinear, especially after leaving violent households. Kiyoko described how she was feeling while living in a women’s shelter:

I am not as, I don’t feel like stress like I was before and I don’t feel depressed like I was before, and I am not really scared like I was before. So, it’s like I am moving, I am going from one stage to the other and I am, I feel like I am taking control back of my life little by little. So that’s what I mean by much better. I am not too depressed about the issue, I am taking steps to work on the issue and I am not feeling like I just don’t have a destination. Now I feel like I am doing things you know, to better myself and get out there more. I am working more towards stability.

Kiyoko was working toward regaining housing stability. However, she did not consider herself to be unstably housed. She noted that she was living in an extremely unstable environment with her abusive partner, and to her, moving into the shelter provided her with more stability. She did note that this was not her “final destination,” but she felt like she was working towards achieving stable housing.

## Discussion

Our findings present the definitions of stability using the words of women who have experienced IPV and low-income. These women defined stability in a multifaceted way. We found that traditional definitions of housing stability that view material aspects of stability as the primary indicator of housing stability are insufficient to describe the experiences of these women. Similar to Sylvestre et al.’s (2009) findings, our research suggests that definitions of housing stability must be broad enough to incorporate women’s lived experiences as well as their ability to be independent and exercise personal choice. Specifically, we found that for women who have left violent households, safety, security, family friendly housing, and comfort are embedded in their definitions of a stable home. Our findings indicate a need to use broad measures of housing stability when studying women who have experienced low-income and IPV.

The women in this study reported that safety and comfort are important dimensions of housing stability. Home was conceptualized as a refuge, where a woman could feel safe and calm, build healthy routines and lifestyles, and rebuild her life. Establishing routine and feeling safe was of particular importance to women who had children or dependents living in their households. Moreover being able to live without fear of victimization contributed to their feelings of safety and security. Robinson (2005) similarly found that the concept of home includes a therapeutic element. Our findings reinforce Easthope’s (2004) and Manzo’s (2014) argument that places can hold deep meanings for different individuals and groups. For the women in this study, being stable involved emotional, social and psychological aspects.

As women moved toward stability they began to experience a greater sense of safety, security, routine and comfort. This suggests that they may be experiencing greater ontological security as they are rediscovering self and through beginning the process of establishing stable homes. Herein, Dunn (2013) and Padgett’s(2007) arguments that the home appears to represent a stabilizing element applies. Establishing a home allowed these women to heal, rebuild self-identity, and begin to feel safe. Using Giddens (1991) concept of ontological security, we argue that home, for these women, contributed to the capacity to reestablish routine and control within their lives, which has the potential to contribute to decreased experiences of anxiety.

We have uncovered a gap between women’s definitions of stability and lived experiences. In other words, most women have a clear idea of what they are aiming toward in their perceptions of achieving stable housing, but few have actually experienced adequate housing stability in their adult lives. Many of these women had begun the process of achieving stable or more stable housing. Like Dunn (2013), we argue that these women had thought about and envisioned their future housing environments, suggesting that the concepts of housing and home influenced their narratives or biographies of self. The women described what stable housing looked like to them, regardless of whether or not they had experienced this in their adult lives. In this sense, their idealized views of home and stability colonized their future views of self. Women were able to envision how stable housing feels, define what it means, and explain its influence on their children and dependents. Stable housing became an ideal for women to work toward.

The experiences of the women who participated in our study suggest that in order to successfully leave abusive households, regain or build ontological security, and establish new, stable households, women must have access to strong, supportive social networks. Social and professional support networks were viewed as important to their ability to maintain housing stability. Developing trusting relationships within their communities contributed to their social, emotional, and psychological wellbeing. Relationships were important in women’s definitions of stability. In addition to building trust, which is a key component of ontological security (Giddens 1991), establishing social connections helped women combat feelings of loneliness which made them vulnerable to returning to abusive relationships.

We argue that meaning and experiences of home, expressed by women as involving feelings of safety and security, are important in defining and securing housing stability for these women. Like Shaw (2004) and Padgett (2007), we acknowledge the importance of both hard (material stability) and soft components (emotional stability) of stable housing. In Ontario, Canada, low-income persons leaving violent households are provided with priority access to social housing units. According to the Special Priority Policy Research Taskforce (2012) allowing priority access has generally provided these households with fast access to housing that is considered to be financially affordable. However, in municipalities with long social housing waiting lists, women may experience lengthy wait times. We argue that all persons who have experienced IPV should be provided with quick access to housing that meets the requirements of affordability. Additionally, our findings point to the importance of providing women who leave with access to psychological and social support while they begin the process of generating some stability in their housing and their lives in general. Greater choice in affordable housing, which includes access to a variety of units in different residential areas where women may establish personal feelings of safety, security, control, comfort and community may also assist in helping women build stable homes and in reestablishing a sense of self. Our findings suggest that providing these types of supports may assist women in rebuilding a sense of home, resulting in improved health and overall wellbeing.

## Conclusions

Although research has emerged that measures housing stability in women who have experienced IPV, until now, we have had little qualitative evidence that describes what stability means for these women. Our research illustrates that for these women, stable housing involves both material and emotive components. These findings have important implications for other scholars wishing to study housing stability in this population. These studies should include measures of safety, comfort, independence, and consistency. In developing more comprehensive and meaningful definitions of stability, which include physical elements of the house and emotional and psychological elements of the home, we may begin to work toward developing research that better assesses experiences of housing stability for women who live or have lived in violent households.

Although many of the women in this study had experienced housing instability and were only beginning to achieve stable housing, moving toward stable housing allowed them to envision positive futures. Stable housing was a stabilizing factor for women. This suggests that home may be integral for ontological security in this population. Future research could attempt to establish a quantitative understanding of the relationship between housing stability and ontological security in women who have experienced IPV and low-income.
